# Fuzzy multi-criteria decision-making framework for controlling methane explosions in coal mines

**DOI:** 10.1007/s11356-023-31782-0

**Published:** 2024-01-06

**Authors:** Nilufer Kursunoglu

**Affiliations:** Department of Petroleum and Natural Gas Engineering, Batman University, Batman, Turkey

**Keywords:** Underground coal mining, Accident, Methane, Explosion, Fuzzy AHP, Fuzzy TOPSIS

## Abstract

The structure of underground coal mines is vulnerable to many mishaps because of the challenging conditions of production and the unique features of the earth. These incidents could cause significant financial and production losses for the mines in addition to worker injury, disability, or death. In coal mines, methane explosions are a frequent threat. Establishing a safe work environment requires managing these problems with an accident control method. The current study used the fuzzy TOPSIS and fuzzy AHP techniques for this aim. The framework was used to tackle the four-alternative problem of underground coal mine explosion control method selection. To identify potential risks of a methane explosion, a data gathering survey was conducted as part of the suggested hybrid methodology. The fuzzy AHP was used to compute the fuzzy weights of the hazards. “Improper ventilation system” is ranked highest out of the 34 sub-risk factors. The fuzzy TOPSIS was then utilized to rank the explosion control methods using the weights. To assess the viability of the study’s conclusions, a sensitivity analysis was carried out. The findings indicate that “improving safety technology” and “financial investments” are the best ways to reduce such events. The results additionally indicate that the fuzzy TOPSIS approach in combination with the fuzzy AHP provides a helpful framework for dynamically assessing mine methane explosion accidents.

**Supplementary Information:**

The online version contains supplementary material available at 10.1007/s11356-023-31782-0.

## Introduction

Coal mining is one of the riskiest occupations in the world due to its complex nature. It is risky because the employees have to adapt to working conditions that are constantly changing. Thus, occupational accident risk in coal mines is much higher compared to other industries (Myers et al. 1998; Poplin et al. 2008; Qiaoxiu et al. 2016; Kursunoglu 2023). Accidents, injuries, fatalities, and occupational diseases in underground coal mining can occur during actions such as excavation operations, installation of the equipment, support, transportation, and such accidents often lead to catastrophic results (Samantra et al. 2017). The key types of incidents in coal mining are gas explosions, accidents caused by machinery/equipment, blasting, roof falls, coal/gas outbursts, flooding, and fire. Among these, gas-related accidents constitute the highest proportion of fatalities in Turkey’s coal mining (Dursun 2019). Theoretically, three basic conditions are required for a methane explosion to occur: (1) combustible methane concentration between 5 and 15%; (2) oxygen concentration greater than 12%; and (3) the source of fire, such as a flame, spontaneous coal combustion, an electric spark, blasting, a hot metal surface, or a temperature above 540 °C. These three circumstances must exist simultaneously. Generally, sufficient oxygen is commonly available since its concentration is usually higher than 20% in a ventilated underground mine (McPherson 1993; Fan et al. 2011; MSHA 2011). A methane explosion is the most catastrophic hazard in coal mining and an issue that needs more attention. It exhibits a severe safety risk for the worldwide coal mining industry. There are still numerous safety issues that need to be resolved, despite the fact that the number of injuries and fatalities caused on by methane explosions is gradually declining. In order to increase the safety of the mine environment, a thorough risk assessment is therefore a necessity to identify the root causes of methane explosion accidents and minimize potential threats (Cheng 2018).

The process of risk assessment should be viewed as more than just a legal requirement. Encouraging all staff members to participate in the risk assessment process will help it accomplish its goals and boost output. In the mining industry, identifying and evaluating present risks are becoming more and more crucial to risk assessment procedures (Samantra et al. 2017; Mutlu and Kalkan 2023). One of the most crucial phases of the occupational health and safety management system is risk assessment. By taking a proactive approach, a risk assessment aims to create a productive, safe, and healthy work environment. Risk assessment is a systematic process that locates all potential risks both inside and outside the workplace. It evaluates the risks associated with hazards, maintains these risks at an acceptable level, and protects against hazards that could impair production, the environment, or workers (Danish and Onder 2020; Mutlu and Sari 2022). Traditional approaches for assessing risk, including as fault-tree analysis, decision-matrix analysis, failure mode and effects analysis, and Fine Kinney, each have advantages and weaknesses. Due to uncertainty or inadequate data, these methods may not always produce satisfactory results. Experts frequently struggle to assign a precise rating to a risk. Additionally, they require a lot of experience. Although the risk factors are meant to be independent, in reality they frequently interact with one another (Ala and Tripathy 2016; Gul and Celik 2018). The limitations of traditional methods can sometimes be overcome by multi-criteria decision making (MCDM) techniques. Recently, MCDM techniques have been employed to help decision-makers rate the risks and also offer a substantial tool to lower the risks to an acceptable level (Klinke and Renn 2002). Additionally, in MCDM approaches, it can be challenging for decision-makers to rate an alternative precisely in terms of the criteria. One benefit of fuzzy MCDM approaches is that fuzzy numbers, rather than crisp numbers, are used to indicate the relative relevance of criteria (Rezaei et al. 2011; Liu et al. 2018). In this study, the most suitable explosion control method for coal mines was chosen using fuzzy MCDM approaches.

As MCDM and fuzzy MCDM-based systems have the potential to solve real world problems with many, competing, and incommensurate criteria while also taking into account human decision-making, they are frequently used to risk assessment challenges in a variety of industries. Traditional risk assessment techniques have a variety of drawbacks, but they continue to be prominent and widely used. Their ability to be used with other tools and ease of use both contribute to their growing popularity. The most extensively used MCDM methodology that combines fuzzy logic and the analytic hierarchy process (AHP) is called the fuzzy analytic hierarchy process (FAHP). The FAHP is suggested in place of the classic AHP to answer hierarchical problems under fuzziness and ambiguity because the conventional AHP cannot exhibit a subjective thinking way. It can help decision-makers get more accurate outcomes and is regarded as a great tool for dealing with qualitative evaluations by employing fuzzy numbers rather than crisp data. The analyst is able to: (1) ensure a precise hazard rating; (2) use group decision-making in evaluating hazards; (3) give relative importance among the risks by pair-wise comparison; and (4) use linguistic variables for the risk factor assessment by using the FAHP in weighting the risk factors of such methods (Gul et al. 2017). The technique for order preference by similarity to ideal solution (TOPSIS) is a strong MCDM technique that was created to find the optimum option based on the principles of the compromise solution. The compromise option can be thought of as selecting the alternative that is both the furthest away from the negative ideal limit and the closest to the ideal limit. Since evaluated evaluations typically correspond to subjective uncertainty, it makes sense to expand TOPSIS to take the problem of fuzzy numbers into consideration (Gul and Guneri 2016). The TOPSIS and fuzzy TOPSIS (FTOPSIS) are two of the most used risk assessment tools. Due to its compromise solutions, a thorough evaluation of both the TOPSIS- and the FTOPSIS-based risk assessment methods reveals that they are often utilized by scholars. Generally, the TOPSIS/FTOPSIS is used to prioritize hazards and related risks. In the pre-evaluation of hazards to assess the hazard parameters, they can also be hybridized with other MCDM approaches such as Fuzzy entropy, Delphi, AHP, and FAHP. The incorporation of advanced technologies, particularly machine learning in safety methodologies extends beyond coal mining and has shown significant promise in enhancing safety measures across various high-risk industries. Similarly, the integration of decision support systems and machine learning approaches has proven to be instrumental in predicting mine fire levels, as demonstrated in the literature (Shahani et al. 2021; Kamran and Shahani 2022; Kamran et al. 2023). The parallels between these advancements and the current research on methane explosion control in coal mines highlight the interdisciplinary nature of safety enhancement efforts. This interdisciplinary approach not only fosters a deeper understanding of the complex dynamics at play in various hazardous environments but also emphasizes the need for a holistic safety strategy. So, in this study, the first stage involved identifying the hazards that led to methane explosions. The FAHP approach was used to prioritize and weight these criteria. The FTOPSIS algorithm and weights derived from the FAHP were combined to rank the four explosion control methods (ECMs) for underground coal mines.

Recent studies on coal mine methane explosions have utilized the methods such as historical data, statistical analysis, fuzzy AHP, Bayesian networks, and FTA (Doyle 2001; Fan et al. 2011; Yin et al. 2017; Tong et al. 2018; Meng et al. 2019; Zhu et al. 2019; Li et al. 2020). However, up until now, there has not been a combined application of the FAHP and FTOPSIS for controlling methane explosion accidents. This study not only weighs the hazard elements but also provides a mechanism for ranking the ECMs. In order to determine effective hazard factors and choose the best ECM to control accidents, the current study integrates the FAHP and FTOPSIS methodologies.

## Significance of the study

The significance of this study lies in its comprehensive approach to addressing the critical issue of methane explosions in underground coal mines. Coal mining remains one of the most perilous occupations globally, with methane explosions posing a severe safety risk. The study recognizes the complexity of the coal mining environment, where employees continually adapt to changing conditions, leading to a higher risk of accidents. The research emphasizes the importance of conducting thorough risk assessments in the coal mining industry, particularly focusing on methane explosions. Traditional risk assessment methods, while widely used, have limitations such as uncertainty and the need for extensive data. The study advocates for the adoption of the MCDM techniques, specifically employing the FAHP and the FTOPSIS.

By integrating the FAHP and the FTOPSIS, the study introduces a novel approach to prioritize hazard elements and rank ECMs. This methodology not only considers the hazards leading to methane explosions but also provides a mechanism for selecting the most effective ECMs. The application of FAHP allows for precise hazard rating, group decision-making, relative importance assessment, and the use of linguistic variables in risk factor assessment. The research addresses a gap in the current literature by combining the FAHP and the FTOPSIS for controlling methane explosion accidents in coal mines. While previous studies have utilized various methods, the integrated application of the FAHP and the FTOPSIS provides a more holistic and systematic approach to hazard prioritization and ECM selection. This study contributes valuable insights to enhance safety measures in the coal mining industry, ultimately minimizing the occurrence and impact of methane explosions.

## Methodology

### Fuzzy analytic hierarchy process

The steps in Buckley’s geometric mean approach are as follows (Emrouznejad and Ho 2018):

- *Step 1*: Forming pairwise comparison matrices
  1 $$\widetilde{\mathrm{D}} \, \mathrm{=} \, \left[\begin{array}{cccc}\mathrm{(1,1,1)}& {\widetilde{\mathrm{a}}}_{12}& \cdots & {\widetilde{\mathrm{a}}}_{\mathrm{1n}}\\ {\widetilde{\mathrm{a}}}_{21}& \mathrm{(1,1,1)}& \cdots & {\widetilde{\mathrm{a}}}_{\mathrm{2n}}\\ \vdots & \vdots & \ddots & \vdots \\ {\widetilde{\mathrm{a}}}_{\mathrm{n1}}& {\widetilde{\mathrm{a}}}_{\mathrm{n2}}& \cdots & \mathrm{(1,1,1)}\end{array}\right]$$
  where $${\widetilde{\mathrm{a}}}_{\mathrm{ij}}{\mathrm{x}}{\widetilde{\mathrm{a}}}_{\mathrm{ji}}\approx {1}$$ and $${\widetilde{\mathrm{a}}}_{\mathrm{ij}} \, \cong \, {\mathrm{w}}_{\mathrm{i}}\mathrm{/}{\mathrm{w}}_{\mathrm{j}}\mathrm{,}$$ *i*, *j* = 1,2, …,*n*
- *Step 2*: Calculation of the fuzzy geometric mean value ${\widetilde{r}}_{i}$, for each criterion *i*
  2 $${\widetilde{\mathrm{r}}}_{\mathrm{i}} \, \mathrm{=} \, {\mathrm{(}{\widetilde{\mathrm{a}}}_{\mathrm{i1}}{\mathrm{x}}{\widetilde{\mathrm{a}}}_{\mathrm{i2}}{\mathrm{x}}\cdots {\widetilde{\mathrm{a}}}_{\mathrm{in}}\mathrm{)}}^{\mathrm{1/}{\mathrm{n}}}$$
- *Step 3*: Calculation of the fuzzy weight ${\widetilde{w}}_{i}$ for each criterion *i*
  3 $${\widetilde{\mathrm{w}}}_{{\mathrm{i}} \, }\mathrm{=} \, {\widetilde{\mathrm{r}}}_{\mathrm{i}}{\mathrm{x}}{\mathrm{(}{\widetilde{\mathrm{r}}}_{1} \, \mathrm{+} \, {\widetilde{\mathrm{r}}}_{2}\mathrm{+}\dots \mathrm{+}{\widetilde{\mathrm{r}}}_{\mathrm{n}}\mathrm{)}}^{-1}$$
  where $${\widetilde{\mathrm{r}}}_{\mathrm{k}} \, \mathrm{=} \, \mathrm{(}{\mathrm{l}}_{\mathrm{k}}\mathrm{, }{\mathrm{m}}_{\mathrm{k}}\mathrm{, }{\mathrm{u}}_{\mathrm{k}}\mathrm{)}$$ and $${{\mathrm{(}\widetilde{\mathrm{r}}}_{\mathrm{k}}\mathrm{)}}^{-1} \, \mathrm{=} \, \mathrm{(1/}{\mathrm{u}}_{\mathrm{k}}\mathrm{, 1/}{\mathrm{m}}_{\mathrm{k}}\mathrm{,1/}{\mathrm{l}}_{\mathrm{k}}\mathrm{)}$$
- *Step 4*: Calculation of the crisp weights. The fuzzy weights ${\widetilde{w}}_{i}=({l}_{i}, {m}_{i}, {u}_{i})$ are defuzzified based on the center of area method.
  4 $${\widetilde{\mathrm{w}}}_{\mathrm{i}} \, \mathrm{=} \, \frac{{\mathrm{l}}_{\mathrm{i}}\mathrm{, }{\mathrm{m}}_{\mathrm{i}}\mathrm{, }{\mathrm{u}}_{\mathrm{i}}}{3}$$

Although fuzzy set theory is a generalization of a crisp set, fuzzy set numbers only include values between 0 and 1. The nonmembership function is denoted by the number 0, while the full membership function is denoted by the number 1. Triangular fuzzy numbers (TFNs) exist in a wide range of forms that can be used in a variety of contexts. Table 1 illustrates the TFNs scoring method, which is frequently used in MCDM issues.

**Table 1:** Fuzzy numbers used in factor comparison (Emrouznejad and Ho 2018)

Linguistic variables	Triangular fuzzy scale	Triangular fuzzy reciprocal scale
Equally significant	(1,1,1)	(1,1,1)
Equally to average significant	(1,2,3)	(1/3,1/2,1)
Averagely significant	(2,3,4)	(1/4,1/3,1/2)
Averagely to strongly significant	(3,4,5)	(1/5,1/4,1/3)
Strongly significant	(4,5,6)	(1/6,1/5,1/4)
Strongly to very strongly significant	(5,6,7)	(1/7,1/6,1/5)
Very strongly significant	(6,7,8)	(1/8,1/7,1/6)
Very strongly to extremely significant	(7,8,9)	(1/9,1/8,1/7)
Extremely significant	(9,9,9)	(1/9,1/9,1/9)

### Fuzzy TOPSIS method

It was first proposed by Hwang and Yoon (1981) and is the most well-known technique for dealing with MCDM problems. The selected alternative should, according to this method, be the farthest from the negative ideal solution and the closest to the positive ideal solution. Chen (2000) created the vertex method to calculate the separation between two TFNs. The following explanation of the FTOPSIS process (Nădăban et al. 2016):

- *Step 1*: Consisting of *K* decision-makers and *A* represents the value of *i*. criterion in a group. In this group, the criteria values of the alternatives are calculated by Eq. (5):
  5 $$\ddot{x} \, \mathrm{=} \, \frac{1}{{\mathrm{K}}}\mathrm{[}{\ddot{x}}_{\mathrm{ij}}^{1}\mathrm{(+}{\ddot{x}}_{\mathrm{ij}}^{2}\left(\mathrm{+}\right)\dots \left(\mathrm{+}\right){\ddot{x}}_{\mathrm{ij}}^{\mathrm{K}}\mathrm{]}$$
  The linguistic values used in the FTOPSIS application and the equivalents of these linguistic values as triangular fuzzy numbers are presented in Table 2.
- *Step 2*: In order to reduce the weights determined by *K* decision-makers for each criterion to a single value, the value of ${\ddot{w}}_{ij}^{k}$ is calculated as in Eq. (6):
  6 $${\ddot{w}}_{\mathrm{j}} \, = \mathrm{ } \frac{1}{{\mathrm{K}}}\mathrm{[}{\ddot{w}}_{\mathrm{j}}^{1}\mathrm{(+}{\ddot{w}}_{\mathrm{j}}^{2}\left(\mathrm{+}\right)\dots \left(\mathrm{+}\right){\ddot{w}}_{\mathrm{j}}^{\mathrm{K}}\mathrm{]}$$
  where *w* indicates the importance of the j. decision-maker.
- *Step 3*: Calculation of fuzzy decision matrix.
  After obtaining a single value for all criteria and alternatives, the decision problem is shown in matrix format as in Eq. (7):
  7 $$\check{D}\mathrm{=} \, \left[\begin{array}{ccc}{\widehat{y}}_{11}& \cdots & {\widehat{y}}_{\mathrm{1n}}\\ \vdots & \ddots & \vdots \\ {\widehat{y}}_{\mathrm{m1}}& \cdots & {\widehat{y}}_{\mathrm{mn}}\end{array}\right]\widehat{W} \, \mathrm{=} \, \mathrm{[}{\widehat{w}}_{1} \, {\widehat{w}}_{2}\dots {\widehat{w}}_{\mathrm{n}}\mathrm{]}$$
  where ${\widehat{y}}_{ij}=\left(\forall i, j\right)$ and ${\widehat{W}}_{j}$ $j=(1, 2,\dots , n)$ are TFNs, *Ď* is fuzzy decision matrix, and *Ŵ* is fuzzy weights matrix.
- *Step 4*: Normalizing the fuzzy decision matrix
  The fuzzy decision matrix is normalized and the normalized fuzzy decision matrix (*Ȓ*) is obtained as in Eq. (8):
  8 $$\widehat{R} \, \mathrm{=} \, {\mathrm{[}{\widehat{r}}_{\mathrm{ij}}\mathrm{]}}_{\mathrm{mxn}}$$
  Benefit (*B*) and cost criteria (*C*) are calculated as below:
  9 $${\widehat{r}}_{\mathrm{ij}} \, \mathrm{=}\left(\frac{{\mathrm{a}}_{\mathrm{ij}}}{{\mathrm{c}}_{\mathrm{j}}}\mathrm{,}\frac{{\mathrm{b}}_{\mathrm{ij}}}{{\mathrm{c}}_{\mathrm{j}}}\mathrm{,}\frac{{\mathrm{c}}_{\mathrm{ij}}}{{\mathrm{c}}_{\mathrm{j}}}\right)\mathrm{, }{\mathrm{j}} \, \in \, {\mathrm{B}}\mathrm{, }{\mathrm{c}}_{\mathrm{j}} \, \mathrm{=} \, \mathrm{max }{\mathrm{c}}_{\mathrm{ij}}\mathrm{, }{\mathrm{j}}\in {\mathrm{B}}$$
  10 $${\widehat{r}}_{{\mathrm{ij}} \, }\mathrm{=}\left(\frac{{\mathrm{a}}_{\mathrm{j}}^{-}}{{\mathrm{c}}_{\mathrm{ij}}}\mathrm{,}\frac{{\mathrm{a}}_{\mathrm{j}}^{-}}{{\mathrm{b}}_{\mathrm{ij}}}\mathrm{,}\frac{{\mathrm{a}}_{\mathrm{j}}^{-}}{{\mathrm{a}}_{\mathrm{ij}}}\right)\mathrm{, }{\mathrm{j}} \, \in \, {\mathrm{C}}\mathrm{, }{\mathrm{a}}_{\mathrm{j}} \, \mathrm{=} \, \mathrm{min }{\mathrm{a}}_{\mathrm{ij}}\mathrm{, }{\mathrm{j}}\in {\mathrm{C}}$$
  where ${\widehat{r}}_{ij}$ and $\left(\forall i, j\right)$ are normalized triangular fuzzy numbers.
- *Step 5*: Calculation of the weighted normalized matrix.
  The weighted normalized fuzzy decision matrix is calculated according to Eqs. (11) and (12), considering the different weights of each decision criterion.
  11 $$\widetilde{V} \, \mathrm{=} \, {\mathrm{[}{\widetilde{v}}_{\mathrm{ij}}\mathrm{]}}_{\mathrm{mxn}} \, {\mathrm{i}} \, \mathrm{=} \, \mathrm{1,2, }\dots \mathrm{, }{\mathrm{m}} \, {\mathrm{j}} \, \mathrm{=} \, \mathrm{1,2, }\cdots \mathrm{, }{\mathrm{n}}$$
  12 $${\widetilde{v}}_{\mathrm{ij}} \, \mathrm{=}{ \, \widehat{r}}_{\mathrm{ij}}\mathrm{(.)}{\widehat{w}}_{\mathrm{j}}$$
- *Step 6*: Calculation of fuzzy positive ideal solution (FPIS) and fuzzy negative ideal solution (FNIS). FPIS and FNIS are calculated as below:
  13 $${\mathrm{A}}^{+} \, \mathrm{=} \, \mathrm{(}{\widetilde{v}}_{1}^{+}\mathrm{, }{\widetilde{v}}_{2}^{+}\mathrm{, }\dots \mathrm{, }{\widetilde{v}}_{\mathrm{n}}^{+}\mathrm{)}$$
  14 $${\mathrm{A}}^{-} \, \mathrm{=} \, \mathrm{(}{\widetilde{v}}_{1}^{-}\mathrm{, }{\widetilde{v}}_{2}^{-}\mathrm{, }\dots \mathrm{, }{\widetilde{v}}_{\mathrm{n}}^{-}\mathrm{)}$$
  where $${\widetilde{v}}_{j}^{+}=\left(\mathrm{1,1},1\right), {\widetilde{v}}_{j}^{-}=\left(\mathrm{0,0},0\right)$$ and $$j=\mathrm{1,2}, \dots , n$$.
- *Step 7*: The distances of each alternative from the positive ideal solution and the negative ideal solution are calculated as below:
  15 $${\mathrm{d}}_{\mathrm{i}}^{+}\mathrm{=}\sum\nolimits_{{\mathrm{j}} \, \mathrm{=} \, {1}}^{\mathrm{n}}{{\mathrm{d}}}_{\mathrm{v}}\left({\widetilde{v}}_{\mathrm{ij}}\mathrm{,}{\widetilde{v}}_{\mathrm{j}}^{+}\right)\mathrm{, }{\mathrm{i}} \, \mathrm{=} \, \mathrm{1,2, }\dots \mathrm{, }{\mathrm{m}}$$
  16 $${\mathrm{d}}_{\mathrm{i}}^{-}\mathrm{=}\sum\nolimits_{{\mathrm{j}} \, \mathrm{=} \, {1}}^{\mathrm{n}}{{\mathrm{d}}}_{\mathrm{v}}\left({\widetilde{v}}_{\mathrm{ij}}\mathrm{,}{\widetilde{v}}_{\mathrm{j}}^{-}\right)\mathrm{, }{\mathrm{i}} \, \mathrm{=} \, \mathrm{1,2,} \, \dots \mathrm{, }{\mathrm{m}}$$
  where ${d}_{v}$ shows the distance between two fuzzy numbers and can be calculated according to Eq. (17):
  17 $${\mathrm{d}}_{\mathrm{v}}\left(\acute{\hat{a}}\mathrm{, }\acute{\hat{e}}\right) \, = \mathrm{ } \sqrt{{\frac{1}{{3}}\mathrm{[}{\mathrm{a}}_{1}-{\mathrm{b}}_{1}\mathrm{]}}^{2}\mathrm{+}{\mathrm{[}{\mathrm{a}}_{2}-{\mathrm{b}}_{2}\mathrm{]}}^{2}\mathrm{+}{\mathrm{[}{\mathrm{a}}_{3}-{\mathrm{b}}_{3}\mathrm{]}}^{2}}$$
- *Step 8*: In order to determine the ranking of the alternatives, the closeness coefficients (CC_i_) for each alternative are calculated. The closeness coefficient of each alternative is calculated by Eq. (18):
  18 $${\mathrm{CC}}_{\mathrm{i}} \, \mathrm{=} \, \frac{{\mathrm{d}}_{\mathrm{i}}^{-}}{{\mathrm{d}}_{\mathrm{i}}^{*}\mathrm{+}{\mathrm{d}}_{\mathrm{i}}^{-}}\mathrm{ , }{\mathrm{i}} \, \mathrm{=} \, \mathrm{1,2, }\dots {\mathrm{m}}$$

**Table 2:** Linguistic variables for alternative ranking (Chen 2000)

Linguistic term	Fuzzy number
Very low (*VL*)	(0,0,1)
Low (*L*)	(0,1,3)
Slightly low (*SL*)	(1,3,5)
Medium (*M*)	(3,5,7)
Slightly good (*SG*)	(5,7,9)
Good (*G*)	(7,9,10)
Very good (*VG*)	(9,10,10)

According to the rank order of CC_i_, the rank of all alternatives can be determined and the best one among the possible alternatives can be selected. The coefficients of convergence take a value between 0 and 1, and the ranking of the alternatives is done with the coefficient of closeness. The large proximity coefficient can be defined as an indicator of the preference of the alternative by the decision-makers.

## Application of fuzzy AHP and fuzzy TOPSIS methodologies

**Fig. 1:**
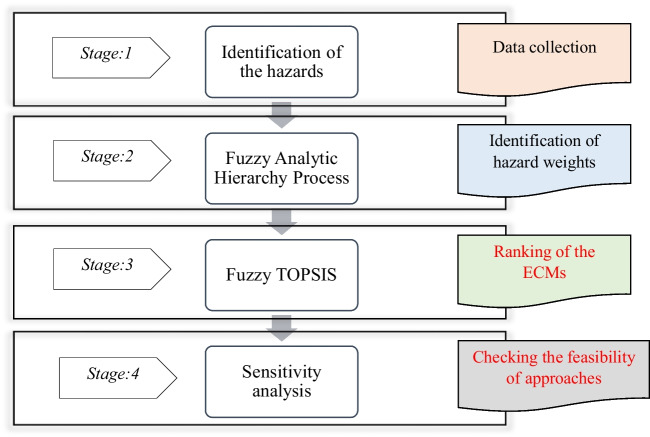
The framework of the study

In order to choose an acceptable ECM for underground coal mines, the current study is divided into four stages. The explanations in the “Methodology” section formed the basis for the analyses. Possible hazards that could have caused the methane explosions were identified in the initial stage. The weights of the hazards were provided in the second step using the FAHP technique, which is effective at controlling uncertainties and permitting flexible calculations. The final part of the process involved ranking the ECMs using the FTOPSIS approach. In order to determine how different criterion weights would have affected alternate ranks in the fourth stage, sensitivity analysis was lastly carried out. The study approach was graphically illustrated in Fig. 1.

### Identification of the explosion hazards

During operation in coal mines, a variety of risks that can result in historical methane explosions are encountered. The accident reports of Turkish Hard Coal Enterprise provided information on the hazards that led to methane explosions at underground coal mines in Turkey (THCE 2019; IU 2022). Data of the study was given in Table 3. To choose the best ECM, 34 hazards were identified. The hazards were divided into eight major categories, including *ventilation design, ventilation system failures, monitoring, equipment, open flame, blasting, planning and atmosphere,* and *human factors*. The main methane explosion events that happened in Turkey between 1947 and 2022 are included in Table 4.

**Table 3:** Hazards of methane explosions

Main hazards	Code	Sub-hazards
Ventilation design (MH_1_)	SH_1_	Insufficient air quantity
	SH_2_	Progressing without control boreholes
	SH_3_	Not applying the provisions of the outburst directive
	SH_4_	Short circuits of ventilation
	SH_5_	Lack of ventilation plan/directive
Ventilation system failures (MH_2_)	SH_6_	Improper ventilation system
	SH_7_	Lack of self-activation in case of failure
	SH_8_	Inappropriate fan position
Monitoring (MH_3_)	SH_9_	Impermanent gas measuring/recording
	SH_10_	Changing sensor locations
	SH_11_	Gas detection system failure
Equipment (MH_4_)	SH_12_	Not to cease electric energy according to gas concentration
	SH_13_	Lack of safety precautions in the electrical devices
	SH_14_	Lack of antistatic materials for pipes, duct system, belts
	SH_15_	Operator failure
	SH_16_	Lack of periodic maintenance and control plan
Open flame (MH_5_)	SH_17_	Fire
	SH_18_	Combustible materials vicinity of the working area
	SH_19_	Welding sparks
	SH_20_	Lack of gas control before/during the welding process
Blasting (MH_6_)	SH_21_	Lack of methane measurements before filling and disposal
	SH_22_	Improper capsule
	SH_23_	Unsuitable explosives (dynamite)
	SH_24_	Ignition by unqualified employees
	SH_25_	Lack of magneto during ignition
Planning and Atmosphere (MH_7_)	SH_26_	Sudden pressure drops
	SH_27_	Over mining
	SH_28_	Inappropriate mining method
	SH_29_	Lack of dust suppression
Human factors (MH_8_)	SH_30_	Insufficient employee
	SH_31_	Insufficient training to employees
	SH_32_	Lack of employees' safety awareness (smoking, lighter)
	SH_33_	Not providing qualified human resources
	SH_34_	Lack of emergency intervention personnel

**Table 4:** Methane explosion accident data (THCE 2019; IU 2022)

Year	Location	Type of mine	Sub-hazards	Fatalities
1942	Armutçuk	Hard coal mine	SH_1_, SH_2_, SH_4_, SH_8_, SH_30_, SH_31_	63
1947	Kozlu	Hard coal mine	SH_5_, SH_12_, SH_14_, SH_15_, SH_28_	53
1954	Kozlu	Hard coal mine	SH_1_, SH_4_, SH_20_, SH_26_, SH_28_	13
1955	Karadon	Hard coal mine	SH_4_, SH_29_, SH_30_, SH_31_, SH_32_	54
1960	Kozlu	Hard coal mine	SH_13_, SH_14_, SH_15_, SH_16_, SH_34_	25
1969	Karadon	Hard coal mine	SH_8_, SH_12_, SH_14_, SH_15_, SH_29_	13
1972	Kozlu	Hard coal mine	SH_26_, SH_28_, SH_30_, SH_32_	16
1975	Karadon	Hard coal mine	SH_1_, SH_4_, SH_8_, SH_16_, SH_29_	13
1975	Karadon	Hard coal mine	SH_15_, SH_16_, SH_28_, SH_29_	13
1978	Armutçuk	Hard coal mine	SH_1_, SH_2_, SH_3_, SH_8_, SH_33_, SH_34_	17
1983	Armutçuk	Hard coal mine	SH_4_, SH_6_, SH_9_, SH_10_, SH_15_, SH_20_, SH_21_	103
1990	Yeni Çeltek	Coal mine	SH_8_, SH_16_, SH_18_, SH_30_, SH_31_, SH_32_, SH_33_	68
1992	Kozlu	Hard coal mine	SH_2_, SH_5_, SH_6_, SH_8_, SH_9_, SH_10_, SH_11_ SH_29_	263
1995	Sorgun	Coal mine	SH_12_, SH_14_, SH_15_, SH_16_, SH_28_, SH_30_	40
2003	Kilimli	Coal mine	SH_4_, SH_15_, SH_21_, SH_22_, SH_26_, SH_31_, SH_32_	4
2003	Ermenek	Coal mine	SH_4_, SH_15_, SH_25_, SH_32_, SH_33_	10
2004	Karadon	Hard coal mine	SH_4_, SH_19_, SH_20_, SH_29_	5
2005	Gediz	Coal mine	SH_28_, SH_30_, SH_31_, SH_34_	18
2006	Dursunbey	Coal mine	SH_18_, SH_31_, SH_32_	17
2009	Mustafakemalpaşa	Coal mine	SH_4_, SH_7_, SH_18_	19
2009	Bükköy	Coal mine	SH_1_, SH_15_, SH_17_, SH_21_, SH_23_, SH_24_, SH_25_,SH_34_	19
2010	Dursunbey	Coal mine	SH_1_, SH_4_, SH_8_, SH_26_, SH_32_, SH_34_	13
2010	Karadon	Hard coal mine	SH_3_, SH1_3_, SH_15_, SH_27_, SH_29_	30
2013	Kozlu	Hard coal mine	SH_6_, SH_2_, SH_3_, SH_22_, SH_30_	8
2022	Amasra	Hard coal mine	SH_2_, SH_6_, SH_9,_ SH_29_, SH_30_	43

### Construction of the Swiss cheese model

**Fig. 2:**
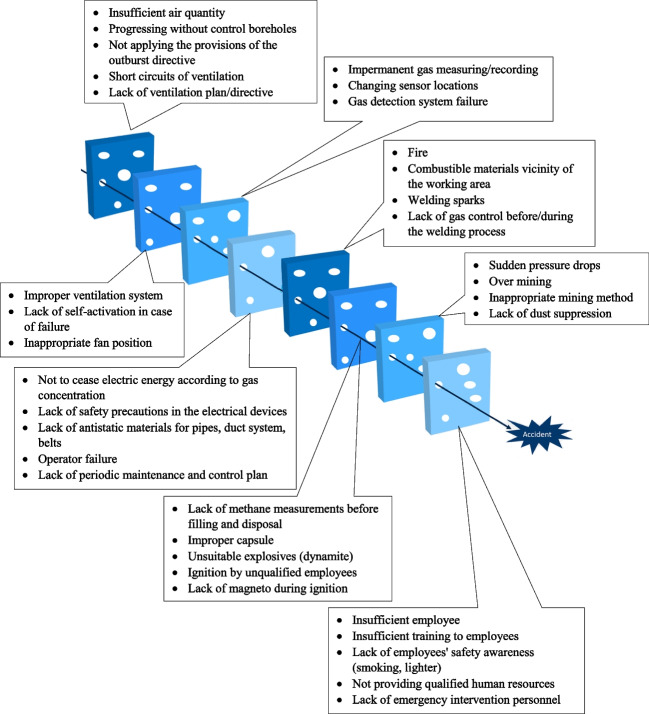
SCM of methane explosion accidents

The problem of human mistake can be viewed from a person or a system perspective. The working environment in this procedure affected the errors. A Swiss cheese model (SCM) diagram was made utilizing the determined causes to demonstrate the role each of these causes contributed. The SCM of accident causation is used to explain the factors that lead to a specific outcome as well as providing a framework for the complete accident investigation process. The causes in different categories in the SCM diagram show the shortcomings in various safety management system levels. One failure feeds into another, or one cause generates another (Reason 2000). According to the SCM framework, Fig. 2 classifies visually the relevant hazard factors of methane explosions that stated in Table 3.

### Application of fuzzy AHP method

**Fig. 3:**
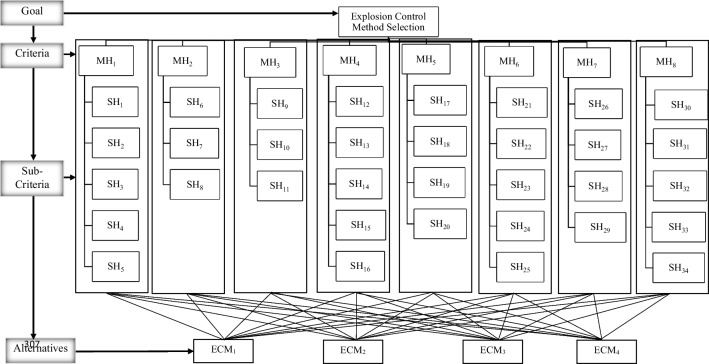
Hierarchical framework of the study

A top-down decision hierarchy is designed as part of the FAHP application. The hierarchy structure begins with an objective (upper level), criteria and sub-criteria (middle level), and alternatives (bottom level). Hierarchical framework of the study was given in Fig. 3. *Explosion control method selection* is specified at the top of the structure (objective). The framework has 34 sub-criteria and four alternatives. The FAHP approach is implemented to define the weights of the hazard factors.

In the process of deciding on an ECM selection, each alternative was evaluated by examining the literature review (Kissell et al. 2007; Lirong et al. 2011; Mahdevari et al. 2014; Wang et al. 2014; Shahani et al. 2019; Ray et al. 2022; Zhang et al. 2022), preventive legislations and reports (NIOSH 2006; ILO 2009; CTEA 2021), and expert team opinion. The alternatives suggested in respect of these considerations were listed as follows:

- ECM_1_: High mining technology
- ECM_2_: Financial investment
- ECM_3_: Application of constructive measures
- ECM_4_: Improving safety technology

The TFNs shown in Table 1 were used to create pairwise comparison matrices and conduct these comparisons in order to obtain the factor weights. To create the pairwise comparison matrices, a group of three experts was consulted. Table 5 displays the experts’ comprehensive information. The judgments were reached through a process of collective decision-making. The arithmetic mean method was used to merge the comparison matrices. The pairwise comparison matrices were given in Appendix A.

**Table 5:** Expert descriptions

Expert	Position	Level of education	Years of experience
E-1	Associate	PhD	8
E-2	OHS specialist	PhD	5
E-3	Engineer	Master	5

Based on the theoretical explanation provided in the “Fuzzy analytic hierarchy process” section, the weight calculations for the hazards were carried out. The geometric mean of each row’s fuzzy numbers is calculated first. The fuzzy weight is then determined by dividing each geometric mean by the total geometric means. Every fuzzy weight is then normalized. The main hazard results are shown in Table 6. The relative weights are derived in a manner similar to that used to create pairwise comparisons. The final sub-hazard weights are computed by multiplying each major hazard weight by each sub-hazard weight (Table 7).

**Table 6:** Main hazard fuzzy weights

Main hazards	Fuzzy weights	Crisp weights	Normalized weights
MH_1_	(0.134 0.272 0.505)	0.304	0.266
MH_2_	(0.110 0.213 0.398)	0.241	0.211
MH_3_	(0.066 0.128 0.243)	0.146	0.128
MH_4_	(0.068 0.122 0.220)	0.137	0.120
MH_5_	(0.042 0.081 0.157)	0.094	0.082
MH_6_	(0.037 0.071 0.152)	0.087	0.077
MH_7_	(0.033 0.059 0.123)	0.072	0.063
MH_8_	(0.027 0.048 0.105)	0.060	0.053

**Table 7:** Final weights of sub-hazards

Main factors	Weight	Sub-hazards	Local weight	Global weight	Rank
MH_1_	0.266	SH_1_	0.404	0.107	2
		SH_2_	0.254	0.067	3
		SH_3_	0.159	0.042	8
		SH_4_	0.127	0.034	11
		SH_5_	0.056	0.015	19
MH_2_	0.211	SH_6_	0.684	0.144	1
		SH_7_	0.210	0.044	7
		SH_8_	0.106	0.022	16
MH_3_	0.128	SH_9_	0.512	0.066	4
		SH_10_	0.371	0.048	6
		SH_11_	0.117	0.015	20
MH_4_	0.120	SH_12_	0.423	0.051	5
		SH_13_	0.235	0.028	12
		SH_14_	0.174	0.021	17
		SH_15_	0.115	0.014	21
		SH_16_	0.053	0.006	31
MH_5_	0.082	SH_17_	0.469	0.039	9
		SH_18_	0.309	0.025	14
		SH_19_	0.123	0.010	26
		SH_20_	0.099	0.008	27
MH_6_	0.077	SH_21_	0.465	0.036	10
		SH_22_	0.177	0.014	22
		SH_23_	0.171	0.013	24
		SH_24_	0.103	0.008	29
		SH_25_	0.085	0.007	30
MH_7_	0.063	SH_26_	0.434	0.027	13
		SH_27_	0.266	0.017	18
		SH_28_	0.212	0.013	23
		SH_29_	0.089	0.006	33
MH_8_	0.053	SH_30_	0.447	0.024	15
		SH_31_	0.214	0.011	25
		SH_32_	0.149	0.008	28
		SH_33_	0.120	0.006	32
		SH_34_	0.069	0.004	34

Table 8 presents fuzzy weights of the sub-hazards. The FAHP method assisted in calculating the hazard weights and the obtained weights were utilized in the FTOPSIS method. The coal mines need to decide on an ECM to mitigate the accidents or to reduce their consequences.

**Table 8:** Fuzzy weights of sub-hazards

Sub-hazards	Fuzzy weights	Sub-hazards	Fuzzy weights
SH_1_	(0.246 0.425 0.701)	SH_18_	(0.197 0.329 0.555)
SH_2_	(0.157 0.270 0.434)	SH_19_	(0.076 0.131 0.220)
SH_3_	(0.105 0.167 0.266)	SH_20_	(0.061 0.100 0.185)
SH_4_	(0.080 0.129 0.222)	SH_21_	(0.263 0.445 0.723)
SH_5_	(0.040 0.059 0.092)	SH_22_	(0.106 0.171 0.267)
SH_6_	(0.450 0.652 0.926)	SH_23_	(0.100 0.164 0.262)
SH_7_	(0.137 0.199 0.285)	SH_24_	(0.057 0.095 0.164)
SH_8_	(0.071 0.097 0.144)	SH_25_	(0.047 0.075 0.139)
SH_9_	(0.360 0.546 0.798)	SH_26_	(0.299 0.485 0.756)
SH_10_	(0.272 0.387 0.576)	SH_27_	(0.146 0.276 0.520)
SH_11_	(0.089 0.122 0.178)	SH_28_	(0.111 0.208 0.432)
SH_12_	(0.284 0.461 0.727)	SH_29_	(0.051 0.089 0.174)
SH_13_	(0.125 0.242 0.448)	SH_30_	(0.261 0.443 0.723)
SH_14_	(0.092 0.172 0.341)	SH_31_	(0.119 0.210 0.354)
SH_15_	(0.065 0.113 0.220)	SH_32_	(0.080 0.141 0.255)
SH_16_	(0.033 0.054 0.097)	SH_33_	(0.062 0.111 0.209)
SH_17_	(0.323 0.517 0.799)	SH_34_	(0.038 0.063 0.117)

### Application of fuzzy TOPSIS method

Numerous advantages are provided by the FTOPSIS, including the ability to account for uncertainty, highly efficient processing, and easier calculations. When a variety of alternatives and criteria are taken into account, it also shows remarkable effectiveness. The FAHP and the FTOPSIS were therefore combined in order to improve the accuracy of determining alternative ranks. Pairwise comparisons provide a way that is easy to comprehend for dealing with even complicated situations, hence this integrated method was developed in the MCDM hierarchical framework (Vaidya and Kumar 2006). In this context, the FTOPSIS method was used in the current study to prioritize explosion control methods. The question of what alternative is necessary to solve the methane explosion problems in underground coal mines has therefore assumed a great deal of significance. According to the theoretical basis in the “Fuzzy TOPSIS method” section, the FTOPSIS computations to rank the alternatives (ECM_1_, ECM_2_, ECM_3_, and ECM_4_) were carried out. To assess the four ECMs, the three experts used language evaluations (Table 2). Table 9 displays the linguistic assessments of the alternatives by the experts based on each sub-criterion in the hierarchy.

**Table 9:** Expert assessments

Sub-hazards	E-1				E-2				E-3			
	ECM_1_	ECM_2_	ECM_3_	ECM_4_	ECM_1_	ECM_2_	ECM_3_	ECM_4_	ECM_1_	ECM_2_	ECM_3_	ECM_4_
SH_1_	SL	SG	SL	M	SL	SL	SG	M	SL	SG	M	SL
SH_2_	SG	M	SL	SL	G	SG	M	SL	SG	SG	SL	M
SH_3_	M	G	SL	SG	SG	M	SL	SL	SG	SG	M	M
SH_4_	SG	G	SL	SL	SL	M	SG	M	M	SG	VG	SL
SH_5_	M	SL	G	SL	SL	SG	G	SL	M	M	SG	SL
SH_6_	M	SL	SG	M	M	SL	SG	SL	SG	M	VG	M
SH_7_	SL	SG	SL	M	SL	SG	SL	SG	M	M	SG	M
SH_8_	SL	SG	G	SL	SL	M	SG	M	SL	SG	G	SL
SH_9_	SG	SL	M	SG	M	SL	SG	M	SG	SL	M	SG
SH_10_	SL	SL	SG	M	M	SL	SG	SL	SL	SL	M	SG
SH_11_	M	G	SL	M	SL	M	SL	SG	SG	SG	SL	SL
SH_12_	SL	SG	M	SL	M	M	G	SL	M	SG	SG	M
SH_13_	SL	SL	SG	M	SL	SL	M	M	M	SL	SG	SL
SH_14_	G	SG	M	SL	SG	G	M	M	SG	M	SG	SL
SH_15_	M	M	SL	SL	SL	M	SG	SL	SL	SG	G	M
SH_16_	SG	SL	M	M	M	SL	SG	SL	G	M	M	M
SH_17_	SL	SG	SL	SG	M	SG	M	SG	M	SL	SG	SL
SH_18_	G	SG	SG	M	SG	G	G	SL	M	SG	G	SL
SH_19_	SL	G	M	SL	SL	SG	M	M	M	G	M	SG
SH_20_	SG	SL	SL	M	M	SL	SG	M	SG	M	SG	SL
SH_21_	M	SL	SL	SG	SG	SL	SL	SG	G	M	M	M
SH_22_	SL	G	G	SL	M	G	SG	SL	SL	SG	SG	SL
SH_23_	M	SG	SG	SL	SL	M	G	M	SL	SG	M	SL
SH_24_	SL	G	G	M	SL	SG	SG	SL	SL	SG	M	M
SH_25_	M	M	SL	SG	SG	SL	SL	M	M	SG	M	M
SH_26_	G	SG	SG	M	SG	SG	G	M	G	SL	SG	SG
SH_27_	SL	SL	M	M	SL	SL	SG	SL	M	SG	G	SL
SH_28_	M	M	SL	SL	SG	M	SL	SL	SG	SL	M	M
SH_29_	M	SL	G	M	SL	M	SG	SG	SG	SG	M	SG
SH_30_	SG	M	SL	SL	SG	SL	SL	SG	G	SG	M	M
SH_31_	M	SG	G	SL	SL	G	SG	M	SL	SG	G	M
SH_32_	SL	SG	SG	M	M	G	M	SL	M	SG	G	SL
SH_33_	M	SL	SG	M	M	SL	SG	SL	SG	M	VG	SG
SH_34_	M	M	SG	SL	SL	SL	SG	M	SG	SL	VG	SG

If the criterion is the benefit criterion, the fuzzy decision matrix is obtained by dividing the elements in each column containing the elements with the highest value, and if the criterion is the cost criterion, then the elements in each column is divided by the element with the lowest value. Decision-makers presume that all criteria are cost-based when evaluating the alternatives (the less the better). A normalized decision matrix and a weighted normalized decision matrix, respectively, were shown in Appendices B and C. The greatest and lowest values in the columns of each criterion in the weighted normalized fuzzy decision matrix were used to calculate the FPIS and FNIS. Then the values ${d}_{i}^{+}$ and ${d}_{i}^{-}$ were computed. Finally, the *CC*_*i*_ of alternatives were calculated, which give the ranking of the alternatives based on distances. Table 10 shows that ECM_4_, with a value of 0.752, is the most preferred action, followed by ECM_2_, ECM_1_, and ECM_3_.

**Table 10:** Ranking of the alternatives based on the FTOPSIS

Alternatives	*d*_i_^+^	*d*_i_^−^	CC_i_	Rank
ECM_1_	2.798	2.344	0.456	3
ECM_2_	2.139	2.984	0.582	2
ECM_3_	3.449	1.666	0.326	4
ECM_4_	1.292	3.913	0.752	1

### Sensitivity analysis

**Fig. 4:**
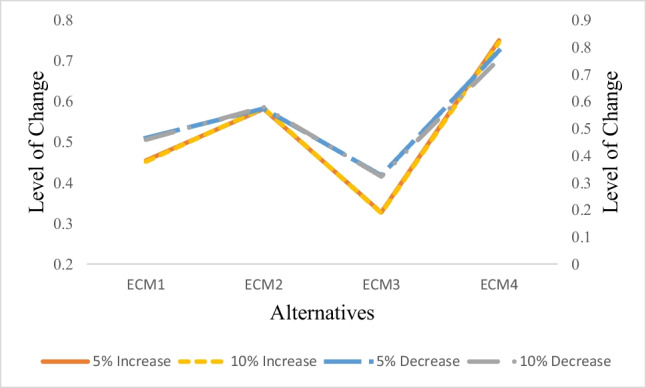
Sensitivity analysis graph

This study included a sensitivity analysis to determine the viability of the results. The primary purpose of the sensitivity analysis is to determine a new ranking of the alternatives by varying the weights of the criteria in various conditions. As a result, the weights of the criteria were changed to compare the final ranks of the alternatives. The weights of the top ten sub-hazards in Table 7 were changed, while the weights of the remaining sub-hazards have remained constant. Table 11 shows the findings of the sensitivity analysis. It may be deduced that the order of alternatives does not vary in the four scenarios. As a result, it was concluded that the results are reliable and efficient. The graphical representation of the sensitivity analysis results is given in Fig. 4.

**Table 11:** Sensitivity analysis results

Level of change	Changed factors	Alternatives	CC_i_	Rank
5% decrease	SH_1_, SH_2_, SH_3_, SH_6_, SH_7_, SH_9_, SH_10_, SH_12_, SH_17_, SH_21_	ECM_1_	0.466	3
		ECM_2_	0.574	2
		ECM_3_	0.328	4
		ECM_4_	0.788	1
5% increase	SH_1_, SH_2_, SH_3_, SH_6_, SH_7_, SH_9_, SH_10_, SH_12_, SH_17_, SH_21_	ECM_1_	0.455	3
		ECM_2_	0.582	2
		ECM_3_	0.327	4
		ECM_4_	0.751	1
10% decrease	SH_1_, SH_2_, SH_3_, SH_6_, SH_7_, SH_9_, SH_10_, SH_12_, SH_17_, SH_21_	ECM_1_	0.459	3
		ECM_2_	0.580	2
		ECM_3_	0.324	4
		ECM_4_	0.757	1
10% increase	SH_1_, SH_2_, SH_3_, SH_6_, SH_7_, SH_9_, SH_10_, SH_12_, SH_17_, SH_21_	ECM_1_	0.452	3
		ECM_2_	0.584	2
		ECM_3_	0.326	4
		ECM_4_	0.746	1

## Results and discussion

### Discussion of the FAHP findings

In this study, the hazard weights were computed using the FAHP method. It focuses on the self-evaluated knowledge of experts using subjective consistency in order to establish the validity of their decision. This method develops the precision of likelihood assessment while shortening the time needed for the fuzzification procedure and the succeeding data processing operation. Due to the high level of uncertainty carried on by limited data and insufficient information, it is hard for field experts to characterize their decision accurately using crisp numbers, that is, by giving a single possibility value to a root event. In these circumstances, it is more practical to provide field experts with a likely range of numerical values stated in linguistic phrases or fuzzy numbers. Fuzzy set theory compromises a methodical tool for dealing with this kind of ambiguity. Following the FAHP, the FTOPSIS was utilized to rank the various ECMs. The study’s expert group’s analysis helped to create a fuzzy assessment matrix utilizing linguistic variables. In this study, normalized decision matrices and weighted normalized fuzzy decision matrices were created (see Appendix section). The manuscript has presented the application of the combination of FAHP and FTOPSIS methods.

**Fig. 5:**
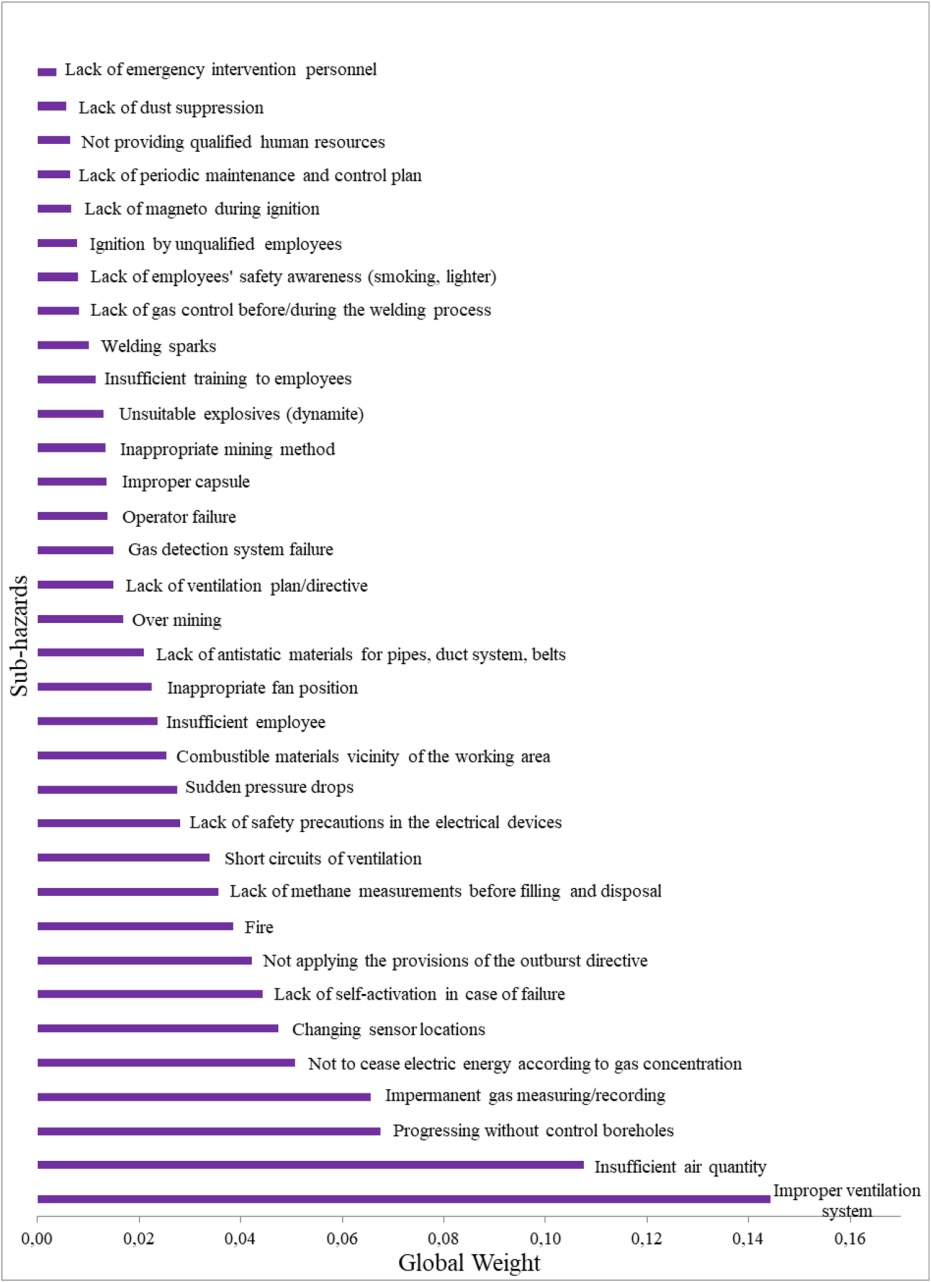
Ranking of sub-hazards

Figure 5 illustrates the ranking of the hazard factors based on the final weights listed in Table 7. Among the 34 sub-risk factors, “improper ventilation system” has the highest ranking, followed by “insufficient air quantity,” which is listed in second place. These two sub-hazard factors’ ultimate weights are 0.144 and 0.107, respectively. It is possible to state that the study’s findings are consistent with the literature. Shi et al. (2018) presented an assessment model of gas explosions based on a fuzzy fault tree analysis. The top four basic actions that have the highest effect on gas explosions are gas error detection or leak detection not found in time, untimely processing, electrical gas welding fire, and an unreasonable ventilation system. Zhu et al. (2019) stated that the causes such as ignition sources and ventilation systems are more substantial in gas explosions. Fan et al. (2011) concluded that the main reasons for mine gas explosion accidents are the gas accumulation originate from ventilation problems and fire. Wang et al. (2014) determined that low-efficiency ventilation and improper ventilation system have a high impact on gas accumulation. Li et al. (2020) conducted a gas explosion risk assessment using fuzzy AHP and Bayesian network. The results indicate that two hazard factors such as ventilation resistance and friction between rocks are most possible to be the direct reasons of the gas explosion. Tong et al. (2018) applied the Bayesian network method to examine the parameters affecting gas explosion accidents. The study showed that gas released from goaf has a minor influence on gas problems concerning gas leakage from the drainage pipe. Poor drainage design for high-gas coal mines is the worst-case leading to severe results.

Providing clean, breathable air for underground workers while simultaneously removing dangerous gases and dusts that production processes release into the atmosphere is the aim of ventilation in underground coal mines. Before coal mines can start operating, an adequate ventilation system must be put in place because life cannot exist without clean, healthy air. Additionally, as underground production areas and the workers rise, the ventilation system needs to be upgraded and its capacity expanded. Methane must be absorbed and removed at its source in order for panel and longwall ventilation systems in coal mines categorized as moderately gaseous to operate as effectively as possible. In most mines, combining ventilation and drainage systems at the same time may be the most effective strategy to maintain low methane levels. If at all possible, it would be much more appropriate to request for drainage prior to coal production or before mining. As a result, operations will be substantially safer when coal production starts since up to 90% of the methane in the seam will have been removed from the environment (Aydın and Kesimal 2007). The efficient implementation of safety requirements must be ensured. Although some coal mines put more emphasis on comprehensiveness of limits than successful execution, the required security performance can be obtained by efficiently executing safety laws. Managers and staff members of coal mining firms must therefore abide by stringent safety legislation, especially those pertaining to safety inspection systems, safety technology advancements, operational safety requirements, safety reward and punishment systems, etc. (Zhang et al. 2022). Additionally, the most recent Turkey accident records were examined (TGIP 2018; Dursun 2019). According to the records, methane explosion occurrences are primarily caused by ventilation problems.

Methane drainage is an essential method for both the efficient use of methane and the prevention of coal mine explosions. Methane drainage is carried out either prior to mining (pre-drainage) or following mining (post-drainage). To lower carbon emissions and provide secure mining conditions, an effective gas drainage system needs to be designed. One of the more common pre-drainage methods for methane is hydraulic fracturing; however, it has the disadvantage of absorbing fracturing fluid in coal seams. The amount of gas that enters the mine ventilation system is reduced by methane drainage. Methane drainage is a pre-excavation mining management technique that is regarded as a significant methane explosion prevention technology. Additionally, reducing the chance of methane explosions is drainage. One of the most important safety precautions to prevent the mine’s gas concentration from rising quickly is the presence of control boreholes. Ventilation systems must be built using computer-aided designs, and their viability must be confirmed through research (Ray et al. 2022). The limit values of the gases indicated in Turkish safety laws and regulations (Occupational Health and Safety in Mine Workplaces Regulation and No. 6331 Occupational Health and Safety Law) must be reorganized in order to comply with international safety standards. The law also has to include the limit values for the gases listed in the rules.

While the focus is on coal mines, the principles of the FAHP and the FTOPSIS model can potentially be adapted for risk assessment in other geotechnical environments, such as metal mines, tunnels, or underground storage facilities. The model’s versatility broadens its applicability across different sectors. The model can be integrated with site-specific data, including geological surveys, gas monitoring results, and historical accident records. By incorporating real-time data, the model becomes more dynamic and responsive to changes in geotechnical conditions over time. Geotechnical environments are influenced by external factors such as weather conditions, seismic activity, and geological shifts. The model can be extended to consider these external factors and their impact on the effectiveness of explosion control methods, providing a more holistic risk assessment.

### Discussion of the FTOPSIS findings

Eliminating methane from coal mines is still a crucial step for the associated explosions even though the previous section discusses suggestions. Methane removal can be place prior to, during, and after the production of coal based on various in-seam and surface-to-mine drilling designs (Shi et al. 2017). Methane explosions continue to occur, which indicates that the regulations are inadequate (CTEA 2021). A strong ventilation resistance distribution is beneficial to prevent the leakage of air into enclosed areas, so fewer fires or explosions will occur. If a ventilation system is effective, resistant, and resilient, the airflow is stable and methane gas may be diluted and dispersed properly (McPherson 1993).

**Fig. 6:**
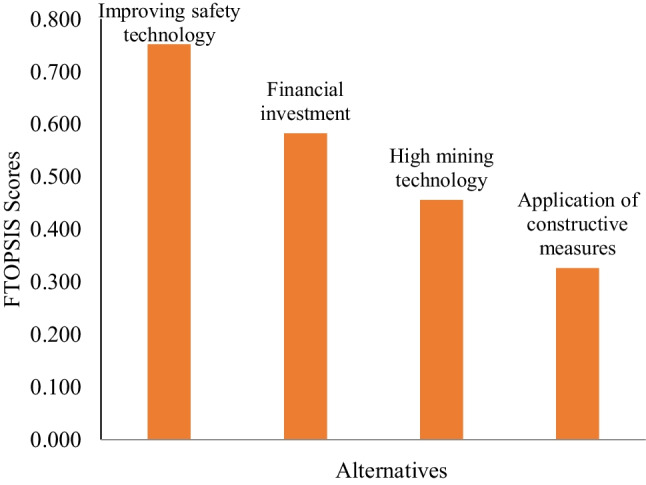
Ranking of the alternatives

The relative weights of the alternatives based on the FTOPSIS were shown in Fig. 6. All alternatives for explosion control in the study can be regarded as substantial. However, it is possible to select ECM_4_ (improving safety technology) as the most important choice, followed by ECM_2_, ECM_1_, and ECM_3_, respectively. More capital should be invested in methane-related equipment. Investments in safety are frequently ignored in some coal mines because it is believed that they will have a negative economic effect. The maintenance of equipment and upgrades, smart ventilation systems, gas monitoring systems, and wireless sensor networks should all receive increased funding from the coal industry. The equipment’s dependability, monitoring accuracy, extraction efficiency, and ventilation stability may all be enhanced as a result. According to Suganthi et al. (2021), long range low power technology (LORA) and wireless underground sensor networks (WUSN) can be used to transmit the monitored parameters from underground to the surface. Lööw et al. (2019) stated that Mining 4.0 will alter more than only the technological environment of mining organizations and workplaces. In a mining operation, the miner is an expert who makes sure that production works smoothly. An operator in Mining 4.0 is not confined to a control room. Instead, the status of the machines and real-time process data accompany the miner as they move about the mine. The miner interacts remotely in multi-competent teams with other operators, subject matter experts, suppliers, and customers to immediately address issues at their source. Even far from the factory, in a “digital twin,” production control might be carried out. In essence, Mining 4.0 imagines an improved miner with technologically enhanced senses and memories. By increasing situational awareness, for instance, using sensors integrated into the operator’s clothing, this technology makes use of and complements human abilities and maintains operational attentiveness. To handle the impacts of heat and lengthy shifts in challenging mine conditions, this could be crucial. ECM_2_ (financial investments) is also essential in comparison to all other possibilities. Financial support for mine producers is important for the growth of mining operations. Incentives should be implemented through mechanisms to increase output and enhance workplace health and safety. A comparative analysis of the present study with existing models from the literature was given in Table 12.

**Table 12:** Comparison of the present study with existing models

Literature	Key parameters	Modeling approach	Validation and reliability	Main findings and ımpacts
Wang et al. (2014)	Fatal gas accidents	Accident reports, statistical analysis	Accident reports from Chinese mines	Identifying main causes of fatal accidents
Shi et al. (2017)	Methane concentration, geological conditions, explosive gases	Mathematical modeling and CFD simulations	Real explosion incident data	Quantitative risk assessment to identify hazardous conditions
Shi et al. (2018)	Gas and dust explosions	Fuzzy logic, fault tree analysis	Mining incident reports and simulations	Assessing complex explosion scenarios with fuzzy logic
Tong et al. (2018)	Mine gas explosion assessment	Bayesian statistics	Mining explosion incident data	Probabilistically determining the risk of mine gas explosions
Lööw et al. (2019)	Impact of new technology from a workplace perspective	IoT, automation, data analytics	Evaluation through worker surveys and industry expert opinions	Advantages and challenges of technological transformation in the workplace
Zhu et al. (2019)	Coal mine fires and explosions	Statistical analysis	Data from mining incidents in China	Determining trends in the frequency of fires and explosions
Li et al. (2020)	Gas explosion risk assessment	Fuzzy analytic hierarchy process, Bayesian networks	Real incidents and expert opinions	Evaluating gas explosion risk considering various factors
Suganthi et al. (2021)	Methane concentration, geological conditions, worker location tracking	Long range (LORA) communication, wireless sensor networks (WUSN)	Field tests in real mining environments and user feedback	Effective system design for worker safety and location tracking
Ray et al. (2022)	Preventive measures for coal mine explosions	Review and analysis	Compliance with mining safety standards in India	Strategies and policies for preventing mine explosions
The present study	Controlling coal methane explosions	Fuzzy AHP, fuzzy TOPSIS	Sensitvity analysis	Selection of the most appropriate explosion control method

## Conclusions

Fatalities and adverse impacts on the environment and mine workers have resulted from methane explosions in underground coal mines. This study identified the hazard factors leading to methane explosions and utilized the FAHP technique to assess their hazard ratings. By employing the FTOPSIS, the optimal explosion control method for underground coal mines was determined. The integration of the FAHP and the FTOPSIS enhances the precision of emergency decision-making, thereby improving the potential effectiveness of strategies for preventing losses in the event of methane explosion incidents. Four solutions were proposed for controlling methane explosion accidents, with the most effective being “improving safety technology.” Sensitivity analysis of the FTOPSIS results was conducted, and the study’s findings align with the results of this analysis. This study represents an innovative examination that combines the selection of an explosion control method with a risk analysis approach. The study’s results suggest an effective methodology for dynamically assessing mine methane explosion events by integrating the FAHP and the FTOPSIS, providing emergency decision-makers with a more accurate evaluation for loss avoidance. It emphasizes that preventing methane accidents goes beyond mere compliance with regulations, urging the adoption of specific hazard factors conformed to each coal mine. For future studies, a comparative analysis could be conducted using other MCDM approaches such as Analytic Network Process (ANP), Vise Kriterijumsa Optimizacija I Kompromisno Resenje (VIKOR), Elemination and Choice Translating Reality English (ELECTRE), and Preference Ranking Organization Method for Enrichment Evaluation (PROMETHEE). Another potential project involves developing a modified sorting MCDM approach based on large-scale group decision-making. Additionally, the creation of a dynamic risk assessment framework integrating real-time data and continuous monitoring, utilizing Internet of Things (IoT) devices, sensors, and data analytics, could provide a more proactive and responsive mitigation strategy. Exploring a modified sorting MCDM approach based on large-scale group decision-making is another potential future project, offering a more comprehensive perspective.

### Supplementary Information

Below is the link to the electronic supplementary material.

Supplementary file1 (DOCX 45 KB)

## Data Availability

The author provided data used in the study in the Appendix section.

## Abbreviations

*AHP*: Analytic hierarchy process
*CC*_*i*_: Closeness coefficients
${d}_{i}^{+}$: Distances of each alternative from the positive ideal solution
${d}_{i}^{-}$: Distances of each alternative from the negative ideal solution
*ECM*: Explosion control method
*FAHP*: Fuzzy analytic hierarchy process
*FNIS*: Fuzzy negative ideal solution
*FPIS*: Fuzzy positive ideal solution
*FTOPSIS*: Fuzzy TOPSIS
*MCDM*: Multi-criteria decision making
*SCM*: Swiss cheese model
*TFN*: Triangular fuzzy number
