# Supplementary material for: Fuzzy multi-criteria decision-making framework for controlling methane explosions in coal mines
Source: Environ Sci Pollut Res Int. 2024 Jan 6;31(6):9045–61. doi: 10.1007/s11356-023-31782-0 (PMC10824880; doi:10.1007/s11356-023-31782-0)
Supplement: Supplementary file 1 — Supplementary file1 (DOCX 45 KB) [file 11356_2023_31782_MOESM1_ESM.docx]

**Appendix A.**

Pair-wise comparison matrices of the FAHP

|  | SH_1_ | SH_2_ | SH_3_ | SH_4_ | SH_5_ |
| --- | --- | --- | --- | --- | --- |
| SH_1_ | (1,1,1) | (0.667,2.667,3.667) | (2,3,4) | (2.333,3.333,4.333) | (4,5,6) |
| SH_2_ | (0.273,0.375,0.600) | (1,1,1) | (2,3,4) | (1,2,3) | (3,4,5) |
| SH_3_ | (0.250,0.333,0.500) | (0.250,0.333,0.500) | (1,1,1) | (1.333,2.000,2.667) | (2.667,3.667,4.667) |
| SH_4_ | (0.231,0.300,0.429) | (0.333,0.500,1.000) | (0.375,0.500,0.750) | 1,1,1) | (2,3,4) |
| SH_5_ | (0.167,0.200,0.250) | (0.200,0.250,0.333) | (0.214,0.273,0.375) | (0.250,0.333,0.500) | (1,1,1) |

|  | SH_6_ | SH_7_ | SH_8_ |
| --- | --- | --- | --- |
| SH_6_ | (1,1,1) | (2.667,3.667,4.667) | (3.333,4.333,5.333) |
| SH_7_ | (0.214,0.273,0.375) | (1,1,1) | (1.667,2.667,3.667) |
| SH_8_ | (0.188,0.231,0.300) | (0.273,0.375,0.600) | (1,1,1) |

**Appendix A.** (*continue*)

|  | SH_9_ | SH_10_ | SH_11_ |
| --- | --- | --- | --- |
| SH_9_ | (1,1,1) | (1.333,2.000,2.667) | (3,4,5) |
| SH_10_ | (0.375,0.500,0.750) | (1,1,1) | (3,4,5) |
| SH_11_ | (0.200,0.250,0.333) | (0.200,0.250,0.333) | (1,1,1) |

|  | SH_12_ | SH_13_ | SH_14_ | SH_15_ | SH_16_ |
| --- | --- | --- | --- | --- | --- |
| SH_12_ | (1,1,1) | (2.333,3.333,4.333) | (2.333,3.333,4.333) | (4,5,6) | (3.333,4.333,5.333) |
| SH_13_ | (0.231,0.300,0.429) | (1,1,1) | (1,2,3) | (1.333,2.333,3.333) | (3.667,4.667,5.667) |
| SH_14_ | (0.231,0.300,0.429) | (0.333,0.500,1.000) | (1,1,1) | (1.333,2.333,3.333) | (2.333,3.333,4.333) |
| SH_15_ | (0.167,0.200,0.250) | (0.300,0.429,0.750) | (0.300,0.429,0.750) | (1,1,1) | (3,4,5) |
| SH_16_ | (0.188,0.231,0.300) | (0.176,0.214,0.273) | (0.231,0.300,0.429) | (0.200,0.250,0.333) | (1,1,1) |

|  | SH_17_ | SH_18_ | SH_19_ | SH_20_ |
| --- | --- | --- | --- | --- |
| SH_17_ | (1,1,1) | (1.667,2.667,3.667) | (3.333,4.333,5.333) | (3.333,4.333,5.333) |
| SH_18_ | (0.273,0.375,0.600) | (1,1,1) | (3,4,5) | (2,3,4) |
| SH_19_ | (0.188,0.231,0.300) | (0.200,0.250,0.333) | (1,1,1) | (1,2,3) |
| SH_20_ | (0.188,0.231,0.300) | (0.250,0.333,0.500) | (0.333,0.500,1.000) | (1,1,1) |

|  | SH_21_ | SH_22_ | SH_23_ | SH_24_ | SH_25_ |
| --- | --- | --- | --- | --- | --- |
| SH_21_ | (1,1,1) | (2.333,3.333,4.333) | (2.667,3.667,4.667) | (2.333,3.333,4.333) | (2.333,3.333,4.333) |
| SH_22_ | (0.231,0.300,0.429) | (1,1,1) | (1.000,1.333,1.667) | (2.000,2.667,3.333) | (1.000,1.667,2.333) |
| SH_23_ | (0.214,0.273,0.375) | (0.600,0.750,1.000) | (1,1,1) | (1.333,2.333,3.333) | (2,3,4) |
| SH_24_ | (0.231,0.300,0.429) | (0.300,0.375,0.500) | (0.300,0.429,0.750) | (1,1,1) | (1,2,3) |
| SH_25_ | (0.231,0.300,0.429) | (0.429,0.600,1.000) | (0.250,0.333,0.500) | (0.333,0.500,1.000) | (1,1,1) |

|  | SH_26_ | SH_27_ | SH_28_ | SH_29_ |
| --- | --- | --- | --- | --- |
| SH_26_ | (1,1,1) | (2.000,2.667,3.333) | (1.667,2.667,3.667) | (3.333,4.333,5.333) |
| SH_27_ | (0.300,0.375,0.500) | (1,1,1) | (1.000,1.667,2.333) | (2.333,3.333,4.333) |
| SH_28_ | (0.273,0.375,0.600) | (0.429,0.600,1.000) | (1,1,1) | (2,3,4) |
| SH_29_ | (0.188,0.231,0.300) | (0.231,0.300,0.429) | (0.250,0.333,0.500) | (1,1,1) |

|  | SH_30_ | SH_31_ | SH_32_ | SH_33_ | SH_34_ |
| --- | --- | --- | --- | --- | --- |
| SH_30_ | (1,1,1) | (2.667,3.667,4.667) | (2.333,3.333,4.333) | (2,3,4) | (3,4,5) |
| SH_31_ | (0.214,0.273,0.375) | (1,1,1) | (1.333,2.333,3.333) | (1.333,2.000,2.667) | (2.667,3.667,4.667) |
| SH_32_ | (0.231,0.300,0.429) | (0.300,0.375,0.500) | (1,1,1) | (1.000,1.667,2.333) | (2,3,4) |
| SH_33_ | (0.250,0.333,0.500) | (0.375,0.500,0.750) | (0.429,0.600,1.000) | (1,1,1) | (1,2,3) |
| SH_34_ | (0.200,0.250,0.333) | (0.214,0.273,0.375) | (0.250,0.333,0.500) | (0.333,0.500,1.000) | (1,1,1) |

**Appendix A.** (*continue*)

|  | MH_1_ | MH_2_ | MH_3_ | MH_4_ | MH_5_ | MH_6_ | MH_7_ | MH_8_ |
| --- | --- | --- | --- | --- | --- | --- | --- | --- |
| MH_1_ | (1,1,1) | (1.333,2.333,3.333) | (1,2,3) | (1.333,2.333,3.333) | (2,3,4) | (1.667,2.667,3.667) | (3.667,4.667,5.667) | (3.667,4.667,5.667) |
| MH_2_ | (0.300,0.429,0.750) | (1,1,1) | (2.333,3.333,4.333) | (3,4,5) | (2,3,4) | (2,3,4) | (1.333,2.333,3.333) | (1.333,2.333,3.333) |
| MH_3_ | (0.333,0.500,1.000) | (0.231,0.300,0.429) | (1,1,1) | (1,1,1) | (2,3,4) | (1.667,2.667,3.667) | (1,2,3) | (1,2,3) |
| MH_4_ | (0.300,0.429,0.750) | (0.200,0.250,0.333) | (1,1,1) | (1,1,1) | (1.667,2.333,3.000) | (1.333,2.333,3.333) | (2.333,3.333,4.333) | (1.000,1.667,2.333) |
| MH_5_ | (0.250,0.333,0.500) | (0.250,0.333,0.500) | (0.250,0.333,0.500) | (0.333,0.500,1.000) | (1,1,1) | (1,2,3) | (1.333,2.333,3.333) | (1.000,1.667,2.333) |
| MH_6_ | (0.273,0.375,0.600) | (0.250,0.333,0.500) | (0.273,0.375,0.600) | (0.300,0.429,0.750) | (0.333,0.500,1.000) | (1,1,1) | (1.333,2.333,3.333) | (1,2,3) |
| MH_7_ | (0.176,0.214,0.273) | (0.300,0.429,0.750) | (0.333,0.500,1.000) | (0.231,0.300,0.429) | (0.300,0.429,0.750) | (0.300,0.429,0.750) | (1,1,1) | (3,4,5) |
| MH_8_ | (0.176,0.214,0.273) | (0.300,0.429,0.750) | (0.333,0.500,1.000) | (0.429,0.600,1.000) | (0.429,0.600,1.000) | (0.333,0.500,1.000) | (0.200,0.250,0.333) | (1,1,1) |

**Appendix B.**

Normalized decision matrix

| Sub-hazards | ECM_1_ | ECM_2_ | ECM_3_ | ECM_4_ |
| --- | --- | --- | --- | --- |
| SH_1_ | (0.200 0.333 1.000) | (0.111 0.176 1.000) | (0.111 0.200 1.000) | (0.142 0.230 1.000) |
| SH_2_ | (0.100 0.130 0.200) | (0.111 0.157 0.333) | (0.142 0.272 1.000) | (0.142 0.272 1.000) |
| SH_3_ | (0.111 0.157 0.333) | (0.100 0.142 0.333) | (0.142 0.272 1.000) | (0.111 0.200 1.000) |
| SH_4_ | (0.111 0.200 1.000) | (0.100 0.142 0.333) | (0.100 0.150 1.000) | (0.142 0.272 1.000) |
| SH_5_ | (0.142 0.230 1.000) | (0.111 0.200 1.000) | (0.100 0.120 0.200) | (0.200 0.333 1.000) |
| SH_6_ | (0.111 0.176 0.333) | (0.142 0.272 1.000) | (0.100 0.125 0.200) | (0.142 0.230 1.000) |
| SH_7_ | (0.142 0.272 1.000) | (0.111 0.157 0.333) | (0.111 0.230 1.000) | (0.111 0.176 0.333) |
| SH_8_ | (0.200 0.333 1.000) | (0.111 0.157 0.333) | (0.100 0.120 0.200) | (0.142 0.272 1.000) |
| SH_9_ | (0.111 0.157 0.333) | (0.200 0.333 1.000) | (0.111 0.176 0.333) | (0.111 0.157 0.333) |
| SH_10_ | (0.142 0.272 1.000) | (0.200 0.333 1.000) | (0.111 0.157 0.333) | (0.111 0.200 1.000) |
| SH_11_ | (0.111 0.200 1.000) | (0.100 0.142 0.333) | (0.200 0.333 1.000) | (0.111 0.200 1.000) |
| SH_12_ | (0.142 0.230 1.000) | (0.111 0.157 0.333) | (0.100 0.142 0.333) | (0.142 0.272 1.000) |
| SH_13_ | (0.142 0.272 1.000) | (0.200 0.333 1.000) | (0.111 0.157 0.333) | (0.142 0.230 1.000) |
| SH_14_ | (0.100 0.130 0.200) | (0.100 0.142 0.333) | (0.111 0.176 0.333) | (0.142 0.272 1.000) |
| SH_15_ | (0.142 0.272 1.000) | (0.111 0.176 0.333) | (0.100 0.157 1.000) | (0.142 0.272 1.000) |
| SH_16_ | (0.100 0.142 0.333) | (0.142 0.272 1.000) | (0.111 0.176 0.333) | (0.142 0.230 1.000) |
| SH_17_ | (0.142 0.230 1.000) | (0.111 0.176 1.000) | (0.111 0.200 1.000) | (0.111 0.176 1.000) |
| SH_18_ | (0.100 0.142 0.333) | (0.100 0.130 0.200) | (0.100 0.120 0.200) | (0.142 0.272 1.000) |
| SH_19_ | (0.142 0.272 1.000) | (0.100 0.120 0.200) | (0.142 0.200 0.333) | (0.111 0.200 1.000) |
| SH_20_ | (0.111 0.157 0.333) | (0.142 0.272 1.000) | (0.111 0.176 1.000) | (0.142 0.230 1.000) |
| SH_21_ | (0.100 0.142 0.333) | (0.142 0.272 1.000) | (0.142 0.272 1.000) | (0.111 0.157 0.333) |
| SH_22_ | (0.142 0.272 1.000) | (0.100 0.120 0.200) | (0.100 0.130 0.200) | (0.200 0.333 1.000) |
| SH_23_ | (0.142 0.272 1.000) | (0.111 0.157 0.333) | (0.100 0.142 0.333) | (0.142 0.272 1.000) |
| SH_24_ | (0.200 0.333 1.000) | (0.100 0.130 0.200) | (0.100 0.142 0.333) | (0.142 0.230 1.000) |
| SH_25_ | (0.111 0.176 0.333) | (0.111 0.200 1.000) | (0.142 0.272 1.000) | (0.111 0.176 0.333) |
| SH_26_ | (0.100 0.120 0.200) | (0.111 0.176 1.000) | (0.100 0.130 0.200) | (0.111 0.176 0.333) |
| SH_27_ | (0.142 0.272 1.000) | (0.111 0.230 1.000) | (0.100 0.142 0.333) | (0.142 0.272 1.000) |
| SH_28_ | (0.111 0.157 0.333) | (0.142 0.230 1.000) | (0.142 0.272 1.000) | (0.142 0.272 1.000) |
| SH_29_ | (0.111 0.200 1.000) | (0.111 0.200 1.000) | (0.100 0.142 0.333) | (0.111 0.157 0.333) |
| SH_30_ | (0.100 0.130 0.200) | (0.111 0.200 1.000) | (0.142 0.272 1.000) | (0.111 0.200 1.000) |
| SH_31_ | (0.142 0.272 1.000) | (0.100 0.130 0.200) | (0.100 0.120 0.200) | (0.142 0.230 1.000) |
| SH_32_ | (0.142 0.230 1.000) | (0.100 0.130 0.200) | (0.100 0.142 0.333) | (0.142 0.272 1.000) |
| SH_33_ | (0.111 0.176 0.333) | (0.142 0.272 1.000) | (0.100 0.125 0.200) | (0.111 0.200 1.000) |
| SH_34_ | (0.111 0.200 1.000) | (0.142 0.272 1.000) | (0.100 0.125 0.200) | (0.111 0.200 1.000) |

**Appendix C.**

Weighted normalized fuzzy decision matrix

| Sub-hazards | ECM_1_ | ECM_2_ | ECM_3_ | ECM_4_ |
| --- | --- | --- | --- | --- |
| SH_1_ | (0.049 0.141 0.701) | (0.027 0.075 0.701) | (0.027 0.085 0.701) | (0.035 0.098 0.701) |
| SH_2_ | (0.015 0.035 0.086) | (0.017 0.042 0.144) | (0.022 0.073 0.434) | (0.022 0.073 0.434) |
| SH_3_ | (0.011 0.026 0.088) | (0.010 0.023 0.088) | (0.015 0.045 0.266) | (0.011 0.033 0.266) |
| SH_4_ | (0.008 0.025 0.222) | (0.008 0.018 0.074) | (0.008 0.019 0.222) | (0.011 0.035 0.222) |
| SH_5_ | (0.005 0.013 0.092) | (0.004 0.011 0.092) | (0.004 0.007 0.018) | (0.008 0.019 0.092) |
| SH_6_ | (0.050 0.115 0.308) | (0.064 0.177 0.926) | (0.045 0.081 0.185) | (0.064 0.150 0.926) |
| SH_7_ | (0.019 0.054 0.285) | (0.015 0.031 0.095) | (0.015 0.045 0.285) | (0.015 0.035 0.095) |
| SH_8_ | (0.014 0.032 0.144) | (0.007 0.015 0.048) | (0.007 0.011 0.028) | (0.010 0.026 0.144) |
| SH_9_ | (0.040 0.086 0.266) | (0.072 0.182 0.798) | (0.040 0.096 0.266) | (0.040 0.086 0.266) |
| SH_10_ | (0.038 0.105 0.576) | (0.054 0.129 0.576) | (0.030 0.061 0.192) | (0.030 0.077 0.576) |
| SH_11_ | (0.009 0.024 0.178) | (0.008 0.017 0.059) | (0.017 0.040 0.178) | (0.009 0.024 0.178) |
| SH_12_ | (0.040 0.106 0.727) | (0.031 0.072 0.242) | (0.028 0.065 0.242) | (0.040 0.125 0.727) |
| SH_13_ | (0.017 0.066 0.448) | (0.025 0.080 0.448) | (0.013 0.038 0.149) | (0.017 0.056 0.448) |
| SH_14_ | (0.009 0.022 0.068) | (0.009 0.024 0.113) | (0.010 0.030 0.113) | (0.013 0.046 0.341) |
| SH_15_ | (0.009 0.031 0.220) | (0.007 0.020 0.073) | (0.006 0.017 0.220) | (0.009 0.031 0.220) |
| SH_16_ | (0.003 0.007 0.032) | (0.004 0.014 0.097) | (0.003 0.009 0.032) | (0.004 0.012 0.097) |
| SH_17_ | (0.046 0.119 0.799) | (0.035 0.091 0.799) | (0.035 0.103 0.799) | (0.035 0.091 0.799) |
| SH_18_ | (0.019 0.047 0.185) | (0.019 0.042 0.111) | (0.019 0.039 0.111) | (0.028 0.089 0.555) |
| SH_19_ | (0.010 0.035 0.220) | (0.007 0.015 0.044) | (0.010 0.026 0.073) | (0.008 0.026 0.220) |
| SH_20_ | (0.006 0.015 0.061) | (0.008 0.027 0.185) | (0.006 0.017 0.185) | (0.008 0.023 0.185) |
| SH_21_ | (0.026 0.063 0.241) | (0.037 0.121 0.723) | (0.037 0.121 0.723) | (0.029 0.070 0.241) |
| SH_22_ | (0.015 0.046 0.267) | (0.010 0.020 0.053) | (0.010 0.022 0.053) | (0.021 0.057 0.267) |
| SH_23_ | (0.014 0.044 0.262) | (0.011 0.025 0.087) | (0.010 0.023 0.087) | (0.014 0.044 0.262) |
| SH_24_ | (0.011 0.031 0.164) | (0.005 0.012 0.032) | (0.005 0.013 0.054) | (0.008 0.022 0.164) |
| SH_25_ | (0.005 0.013 0.046) | (0.005 0.015 0.139) | (0.006 0.020 0.139) | (0.005 0.013 0.046) |
| SH_26_ | (0.029 0.058 0.151) | (0.033 0.085 0.756) | (0.029 0.063 0.151) | (0.033 0.085 0.252) |
| SH_27_ | (0.020 0.075 0.520) | (0.016 0.063 0.520) | (0.014 0.039 0.173) | (0.020 0.075 0.520) |
| SH_28_ | (0.012 0.032 0.144) | (0.015 0.048 0.432) | (0.015 0.056 0.432) | (0.015 0.056 0.432) |
| SH_29_ | (0.005 0.017 0.174) | (0.005 0.017 0.174) | (0.005 0.012 0.058) | (0.005 0.014 0.058) |
| SH_30_ | (0.026 0.057 0.144) | (0.029 0.088 0.723) | (0.037 0.120 0.723) | (0.029 0.088 0.723) |
| SH_31_ | (0.017 0.057 0.354) | (0.011 0.027 0.070) | (0.011 0.025 0.070) | (0.017 0.048 0.354) |
| SH_32_ | (0.011 0.032 0.255) | (0.008 0.018 0.051) | (0.008 0.020 0.085) | (0.011 0.038 0.255) |
| SH_33_ | (0.006 0.019 0.069) | (0.008 0.030 0.209) | (0.006 0.013 0.041) | (0.006 0.022 0.209) |
| SH_34_ | (0.004 0.012 0.117) | (0.005 0.017 0.117) | (0.003 0.007 0.023) | (0.004 0.012 0.117) |
